# Bis[benzyl *N*′-(3-phenyl­prop-2-enyl­idene)hydrazinecarbodithio­ato-κ^2^ *N*′,*S*]zinc(II)

**DOI:** 10.1107/S1600536808005643

**Published:** 2008-03-05

**Authors:** Hoong-Kun Fun, Suchada Chantrapromma, M. T. H. Tarafder, M. Toihidul Islam, C. M. Zakaria, M. A. A. A. A. Islam

**Affiliations:** a X-ray Crystallography Unit, School of Physics, Universiti Sains Malaysia, 11800 USM, Penang, Malaysia; b Department of Chemistry, Faculty of Science, Prince of Songkla University, Hat-Yai, Songkhla 90112, Thailand; c Department of Chemistry, Rajshahi University, Rajshahi 6205, Bangladesh; d Department of Chemistry, Rajshahi University of Engineering and Technology, Rajshahi 6205, Bangladesh

## Abstract

In the title Zn^II^ complex, [Zn(C_17_H_15_N_2_S_2_)_2_], the Zn^II^ atom lies on a twofold rotation axis. It exists in a tetra­hedral geometry, chelated by two deprotonated Schiff base ligands. The dihedral angle between each ligand is 71.48 (8)°. Mol­ecules are connected by weak C—H⋯S inter­molecular inter­actions into chains along the *c* axis. The crystal structure is further stabilized by C—H⋯π inter­actions involving the phenyl ring of the 3-phenyl­prop-2-enyl­idene unit.

## Related literature

For the synthesis and structure of *S*-benzyl­dithio­carbaza­tes, see: Ali & Tarafder (1977 ▶); Shanmuga Sundara Raj *et al.* (2000 ▶). For the structures of Zn^II^ complexes, see: Latheef *et al.* (2007 ▶); Tarafder, Chew *et al.* (2002 ▶). For the structures of other metal dithio­carbaza­tes, see: Ali *et al.* (2001 ▶, 2002 ▶, 2008 ▶); Chew *et al.* (2004 ▶); Crouse *et al.* (2004 ▶); Tarafder *et al.* (2001 ▶, 2008 ▶); Tarafder, Chew *et al.* (2002 ▶); Tarafder, Jin *et al.* (2002 ▶). For the bioactivity of metal *S*-benzyl­dithio­carbaza­tes, see, for example: Ali *et al.* (2001 ▶, 2002 ▶); Tarafder *et al.* (2001 ▶); Tarafder, Jin *et al.* (2002 ▶).
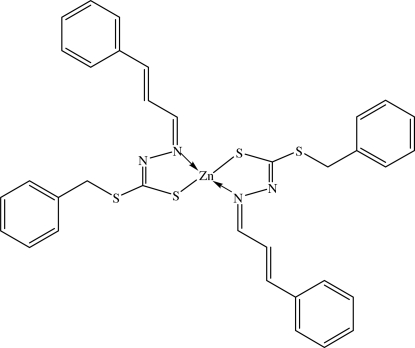


## Experimental

### 

#### Crystal data

[Zn(C_17_H_15_N_2_S_2_)_2_]
*M* *_r_* = 688.23
Orthorhombic, 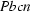

*a* = 36.0897 (4) Å
*b* = 9.9310 (1) Å
*c* = 8.7633 (1) Å
*V* = 3140.83 (6) Å^3^
*Z* = 4
Mo *K*α radiation
μ = 1.08 mm^−1^
*T* = 100.0 (1) K
0.37 × 0.25 × 0.17 mm

#### Data collection

Bruker SMART APEXII CCD area-detector diffractometer
Absorption correction: multi-scan (*SADABS*; Bruker, 2005 ▶) *T*_min_ = 0.692, *T*_max_ = 0.841
82655 measured reflections
4580 independent reflections
4071 reflections with *I* > 2σ(*I*)
*R*_int_ = 0.042

#### Refinement

*R*[*F* ^2^ > 2σ(*F* ^2^)] = 0.028
*wR*(*F* ^2^) = 0.091
*S* = 1.15
4580 reflections
195 parameters
H-atom parameters constrained
Δρ_max_ = 0.49 e Å^−3^
Δρ_min_ = −0.32 e Å^−3^

### 

Data collection: *APEX2* (Bruker, 2005 ▶); cell refinement: *APEX2*; data reduction: *SAINT* (Bruker, 2005 ▶); program(s) used to solve structure: *SHELXTL* (Sheldrick, 2008 ▶); program(s) used to refine structure: *SHELXTL*; molecular graphics: *SHELXTL*; software used to prepare material for publication: *SHELXTL* and *PLATON* (Spek, 2003 ▶).

## Supplementary Material

Crystal structure: contains datablocks global, I. DOI: 10.1107/S1600536808005643/ng2427sup1.cif

Structure factors: contains datablocks I. DOI: 10.1107/S1600536808005643/ng2427Isup2.hkl

Additional supplementary materials:  crystallographic information; 3D view; checkCIF report

## Figures and Tables

**Table 1:** Hydrogen-bond geometry (Å, °)

*D*—H⋯*A*	*D*—H	H⋯*A*	*D*⋯*A*	*D*—H⋯*A*
C13—H13*A*⋯S2^i^	0.93	2.76	3.6697 (17)	167
C11—H11*B*⋯*Cg*1^ii^	0.97	2.98	3.5785 (17)	121

Symmetry codes: (i) 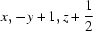; (ii) 
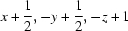
.

**Fig. 1.**
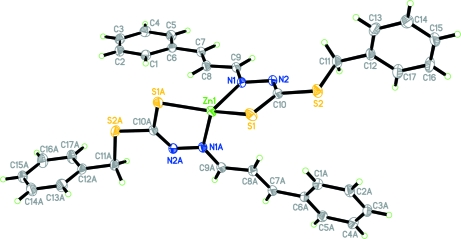
The asymmetric unit of the title compound, showing 50% probability displacement ellipsoids and the atomic numbering. Atoms labelled with suffix A are generated by the symmetry operation (-x, y, 1/2 - z).

**Fig. 2.**
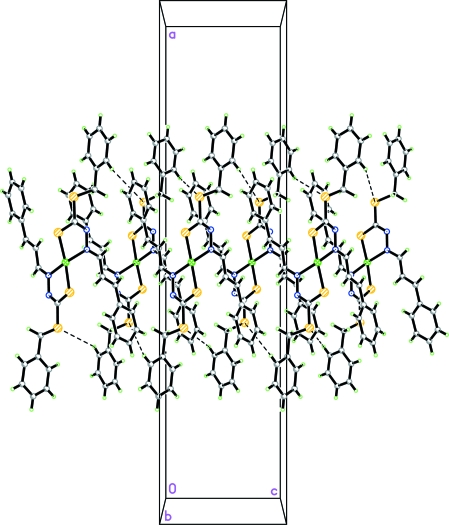
The crystal packing of the title compound, viewed along the b axis. Intermolecular C—H···S weak interactions are shown as dashed lines.

## supplementary crystallographic information

### Comment

The coordination chemistry of the ligands derived from *S*-benzyldithiocarbazate (SBDTC) had been of immense interests because of their intriguing coordination chemistry as well as their increasingly important biomedical properties (Ali *et al.*, 2001; 2002; Tarafder *et al.*, 2001; Tarafder, Jin *et al.*, 2002*b*). Synthesis (Ali & Tarafder, 1977) and structure (Shanmuga Sundara Raj *et al.*, 2000) of SBDTC were reported. We have previously reported the Schiff bases complexes derived from dithiocarbazate derivatives (Ali *et al.*, 2001; 2002; 2008; Chew *et al.*, 2004; Crouse *et al.*, 2004; Tarafder *et al.*, 2001, 2008; Tarafder, Chew *et al.*, 2002; Tarafder, Jin *et al.*, 2002). In continuation of our interests, we report herein the X-ray structure of the zinc(II) complex of Schiff base ligand of SBDTC which is found to be isostructural with the copper(II) analog (Tarafder *et al.*, 2008).

The Zn^II^ atom of the title complex, lies on a twofold rotation axis and therefore the asymmetric unit contains one-half of a molecule (Fig. 1). The ligands coordinate to the Zn^II^ through the two azomethine nitrogen and the two thiolate sulfur atoms forming a distorted tetrahedral geometry (Fig. 1). Both the two nitrogen atoms (N1 and N1A) and two sulfur atoms (S1 and S1A) from the two ligands are coordinated at opposite positions. The NS chelation results in the two five membered Zn^II^-bidentate rings (Zn1, N1, N2, C8, S1), atom Zn1 having a maximum deviation of 0.0839 (5) Å. The dihedral angle between these Zn^II^-bidentate rings is 80.03 (4) °. The smaller angle around Zn^II^ is 86.96 (3) ° for N1—Zn1—S1. The N—Zn—N and S—Zn—S bond angles are 104.32 (7) ° and 134.49 (2) °, respectively. The Zn1—N1 distance of 2.0662 (12) Å is slightly longer compared to the [Zn(C_14_H_18_N_3_OS)_2_] by Latheef *et al.*, 2007 (Zn—N = 2.026 (3) and 2.040 (3) Å) whereas the Zn1—S1 distance of 2.2636 (4) Å in the title complex is in the same range (Zn—S = 2.2597 (13) and 2.2462 (12) Å (Latheef *et al.*, 2007)). The mean plane of the prop-2-enylidene moiety (C7/C8/C9/N1/N2) makes a dihedral angle of 12.25 (12)° with mean plane of the attached C1–C6 phenyl ring. Atoms N1, N2, C10, S1 and S2 lie on the same plane and this plane makes a dihedral angle of 76.75 (6) ° with the C12–C17 phenyl ring. The dihedral angle between the two phenhyl rings (C1–C6 and C12–C17) is 71.48 (8)°. Bond lengths and angles observed in the Schiff base ligand are of normal values.

In the crystal packing (Fig. 2), the molecules are interconnected by weak C—H···S intermolecular interactions (Table 1) into chains along the *c* axis. The crystal is further stabilized by C—H···π interactions (Table 1) involving the C1–C6 phenyl ring (centroid *Cg*_1_) of the 3-phenylprop-2-enylidene moiety.

### Experimental

The Schiff base ligand was prepared following the literature procedure (Tarafder *et al.*, 2008) by adding cinamaldehyde (1.32 g, 10 mmol) to a hot solution of *S*-benzyldithiocarbazate (SBDTC) (1.98 g, 10 mmol) in absolute ethanol (40 ml). The mixture was refluxed for 10 min. The yellow precipitate, which formed, was isolated and washed with hot ethanol. The yellow solid was recrystallized from absolute ethanol (Yield: 1.52 g, 46%). The zinc complex was synthesized by adding the solution of the Schiff base ligand (0.31 g, 1 mmol) in absolute ethanol (70 ml) to a solution of zinc nitrate hexahydrate (0.15 g, 0.5 mmol) in absolute ethanol (5 ml) and stirred under boiling condition for 10 min. A resultant yellow precipitate was separated and washed with hot ethanol (Yield: 0.29 g, 63%). Yellow single crystals of the title complex were crystallized from a mixture solution of chloroform/absolute ethanol (70:5 *v*/*v*) after 40 days at room temperature and further recrystallized from chloroform (40 ml) by slow evaporation at 296 K after 10 days, *M*.p 457–458 K.

### Refinement

All H atoms were positioned geometrically and allowed to ride on their parent atoms, with d(C—H) = 0.93 Å, for CH and aromatic, 0.97 Å, for CH_2_ and *U*_iso_ = 1.2*U*_eq_(C). The highest residual electron density peak is located at 0.60 Å from C1 and the deepest hole is located at 0.54 Å from Zn1.

### Crystal data

**Table:**

[Zn(C_17_H_15_N_2_S_2_)_2_]	*D*_x_ = 1.455 Mg m^−^^3^
*M**_r_* = 688.23	Melting point = 457–458 K
Orthorhombic, *P**b**c**n*	Mo *K*α radiation λ = 0.71073 Å
Hall symbol: -P 2n 2ab	Cell parameters from 4580 reflections
*a* = 36.0897 (4) Å	θ = 1.1–30.0º
*b* = 9.9310 (1) Å	µ = 1.08 mm^−^^1^
*c* = 8.7633 (1) Å	*T* = 100.0 (1) K
*V* = 3140.83 (6) Å^3^	Block, yellow
*Z* = 4	0.37 × 0.25 × 0.17 mm
*F*_000_ = 1424	

### Data collection

**Table:**

Bruker SMART APEXII CCD area-detector diffractometer	4580 independent reflections
Radiation source: fine-focus sealed tube	4071 reflections with *I* > 2σ(*I*)
Monochromator: graphite	*R*_int_ = 0.042
Detector resolution: 8.33 pixels mm^-1^	θ_max_ = 30.0º
*T* = 100.0(1) K	θ_min_ = 1.1º
ω scans	*h* = −50→50
Absorption correction: multi-scan(SADABS; Bruker, 2005)	*k* = −13→13
*T*_min_ = 0.692, *T*_max_ = 0.841	*l* = −12→12
82655 measured reflections	

### Refinement

**Table:**

Refinement on *F*^2^	Secondary atom site location: difference Fourier map
Least-squares matrix: full	Hydrogen site location: inferred from neighbouring sites
*R*[*F*^2^ > 2σ(*F*^2^)] = 0.028	H-atom parameters constrained
*wR*(*F*^2^) = 0.091	*w* = 1/[σ^2^(*F*_o_^2^) + (0.0478*P*)^2^ + 1.4067*P*] where *P* = (*F*_o_^2^ + 2*F*_c_^2^)/3
*S* = 1.15	(Δ/σ)_max_ = 0.001
4580 reflections	Δρ_max_ = 0.49 e Å^−^^3^
195 parameters	Δρ_min_ = −0.32 e Å^−^^3^
Primary atom site location: structure-invariant direct methods	Extinction correction: none

### Special details

**Table:**

Experimental. The low-temparture data was collected with the Oxford Cyrosystem Cobra low-temperature attachment.
Geometry. All e.s.d.'s (except the e.s.d. in the dihedral angle between two l.s. planes) are estimated using the full covariance matrix. The cell e.s.d.'s are taken into account individually in the estimation of e.s.d.'s in distances, angles and torsion angles; correlations between e.s.d.'s in cell parameters are only used when they are defined by crystal symmetry. An approximate (isotropic) treatment of cell e.s.d.'s is used for estimating e.s.d.'s involving l.s. planes.
Refinement. Refinement of *F*^2^ against ALL reflections. The weighted *R*-factor *wR* and goodness of fit *S* are based on *F*^2^, conventional *R*-factors *R* are based on *F*, with *F* set to zero for negative *F*^2^. The threshold expression of *F*^2^ > σ(*F*^2^) is used only for calculating *R*-factors(gt) *etc*. and is not relevant to the choice of reflections for refinement. *R*-factors based on *F*^2^ are statistically about twice as large as those based on *F*, and *R*- factors based on ALL data will be even larger.

### Fractional atomic coordinates and isotropic or equivalent isotropic displacement parameters (Å^2^)

**Table:**

	*x*	*y*	*z*	*U*_iso_*/*U*_eq_	
Zn1	0.0000	0.42216 (2)	0.2500	0.01855 (8)	
S1	−0.056843 (10)	0.51031 (4)	0.20596 (5)	0.02295 (9)	
S2	−0.126368 (10)	0.40453 (4)	0.31408 (5)	0.02541 (10)	
N1	−0.02538 (3)	0.29452 (12)	0.40410 (14)	0.0177 (2)	
N2	−0.06402 (3)	0.29289 (12)	0.40147 (14)	0.0183 (2)	
C1	0.10940 (4)	0.21479 (15)	0.60174 (17)	0.0215 (3)	
H1A	0.1007	0.2854	0.5420	0.026*	
C2	0.14657 (4)	0.20851 (16)	0.63939 (18)	0.0238 (3)	
H2B	0.1627	0.2745	0.6040	0.029*	
C3	0.16001 (4)	0.10408 (18)	0.72987 (18)	0.0251 (3)	
H3A	0.1851	0.0999	0.7541	0.030*	
C4	0.13595 (4)	0.00646 (18)	0.78363 (19)	0.0263 (3)	
H4A	0.1448	−0.0627	0.8451	0.032*	
C5	0.09847 (4)	0.01154 (17)	0.74589 (18)	0.0233 (3)	
H5A	0.0824	−0.0543	0.7825	0.028*	
C6	0.08479 (4)	0.11508 (15)	0.65326 (16)	0.0191 (3)	
C7	0.04530 (4)	0.11779 (15)	0.61642 (17)	0.0197 (3)	
H7A	0.0304	0.0534	0.6627	0.024*	
C8	0.02865 (4)	0.20541 (15)	0.52127 (17)	0.0203 (3)	
H8A	0.0434	0.2660	0.4676	0.024*	
C9	−0.01069 (4)	0.20968 (15)	0.49886 (16)	0.0194 (3)	
H9A	−0.0258	0.1509	0.5530	0.023*	
C10	−0.07830 (4)	0.38814 (15)	0.31855 (16)	0.0188 (3)	
C11	−0.14245 (4)	0.29477 (17)	0.46680 (18)	0.0239 (3)	
H11A	−0.1382	0.2011	0.4407	0.029*	
H11B	−0.1295	0.3146	0.5611	0.029*	
C12	−0.18343 (4)	0.32244 (15)	0.48382 (17)	0.0216 (3)	
C13	−0.19570 (4)	0.42883 (18)	0.5729 (2)	0.0300 (3)	
H13A	−0.1785	0.4831	0.6226	0.036*	
C14	−0.23315 (5)	0.4555 (2)	0.5890 (2)	0.0327 (4)	
H14A	−0.2410	0.5265	0.6503	0.039*	
C15	−0.25893 (4)	0.37653 (18)	0.51415 (19)	0.0281 (3)	
H15A	−0.2841	0.3942	0.5249	0.034*	
C16	−0.24710 (4)	0.27147 (19)	0.4234 (2)	0.0316 (4)	
H16A	−0.2644	0.2187	0.3722	0.038*	
C17	−0.20951 (4)	0.24407 (17)	0.4082 (2)	0.0281 (3)	
H17A	−0.2018	0.1729	0.3470	0.034*	

### Atomic displacement parameters (Å^2^)

**Table:**

	*U*^11^	*U*^22^	*U*^33^	*U*^12^	*U*^13^	*U*^23^
Zn1	0.01629 (12)	0.02017 (13)	0.01920 (13)	0.000	0.00389 (8)	0.000
S1	0.02122 (17)	0.02294 (18)	0.02470 (18)	0.00512 (13)	0.00525 (14)	0.00599 (14)
S2	0.01511 (16)	0.0341 (2)	0.02701 (19)	0.00377 (14)	−0.00046 (13)	0.00913 (15)
N1	0.0135 (5)	0.0205 (5)	0.0191 (5)	0.0002 (4)	0.0008 (4)	−0.0007 (4)
N2	0.0131 (5)	0.0227 (6)	0.0191 (5)	−0.0007 (4)	−0.0003 (4)	0.0002 (5)
C1	0.0198 (6)	0.0222 (7)	0.0226 (7)	0.0018 (5)	−0.0006 (5)	0.0003 (5)
C2	0.0176 (6)	0.0265 (7)	0.0272 (7)	−0.0010 (5)	0.0016 (5)	−0.0024 (6)
C3	0.0168 (6)	0.0325 (8)	0.0260 (7)	0.0047 (6)	−0.0026 (5)	−0.0048 (6)
C4	0.0240 (7)	0.0294 (8)	0.0255 (7)	0.0061 (6)	−0.0039 (6)	0.0025 (6)
C5	0.0214 (7)	0.0241 (7)	0.0243 (7)	0.0016 (6)	−0.0001 (5)	0.0029 (6)
C6	0.0166 (6)	0.0224 (6)	0.0184 (6)	0.0019 (5)	−0.0006 (5)	−0.0011 (5)
C7	0.0163 (6)	0.0228 (7)	0.0199 (6)	−0.0003 (5)	0.0008 (5)	−0.0003 (5)
C8	0.0155 (6)	0.0235 (7)	0.0219 (6)	−0.0004 (5)	0.0011 (5)	0.0008 (5)
C9	0.0164 (6)	0.0226 (7)	0.0192 (6)	−0.0007 (5)	0.0009 (5)	0.0008 (5)
C10	0.0156 (6)	0.0227 (7)	0.0182 (6)	0.0008 (5)	0.0010 (5)	−0.0008 (5)
C11	0.0151 (6)	0.0301 (8)	0.0266 (7)	0.0006 (5)	−0.0003 (5)	0.0060 (6)
C12	0.0148 (6)	0.0268 (7)	0.0230 (7)	0.0001 (5)	−0.0011 (5)	0.0044 (6)
C13	0.0212 (7)	0.0377 (9)	0.0311 (8)	−0.0008 (6)	−0.0057 (6)	−0.0092 (7)
C14	0.0235 (8)	0.0425 (10)	0.0322 (8)	0.0068 (7)	−0.0010 (6)	−0.0106 (8)
C15	0.0153 (6)	0.0392 (9)	0.0297 (8)	0.0025 (6)	0.0009 (6)	0.0018 (7)
C16	0.0172 (7)	0.0347 (9)	0.0428 (10)	−0.0042 (6)	−0.0036 (6)	−0.0053 (7)
C17	0.0189 (7)	0.0273 (8)	0.0380 (9)	−0.0004 (6)	−0.0015 (6)	−0.0070 (7)

### Geometric parameters (Å, °)

**Table:**

Zn1—N1	2.0662 (12)	C6—C7	1.4616 (19)
Zn1—N1^i^	2.0662 (12)	C7—C8	1.347 (2)
Zn1—S1^i^	2.2634 (4)	C7—H7A	0.9300
Zn1—S1	2.2636 (4)	C8—C9	1.4337 (19)
S1—C10	1.7450 (15)	C8—H8A	0.9300
S2—C10	1.7428 (14)	C9—H9A	0.9300
S2—C11	1.8210 (16)	C11—C12	1.5118 (19)
N1—C9	1.2964 (18)	C11—H11A	0.9700
N1—N2	1.3948 (15)	C11—H11B	0.9700
N2—C10	1.2994 (19)	C12—C13	1.386 (2)
C1—C2	1.383 (2)	C12—C17	1.390 (2)
C1—C6	1.405 (2)	C13—C14	1.384 (2)
C1—H1A	0.9300	C13—H13A	0.9300
C2—C3	1.393 (2)	C14—C15	1.382 (2)
C2—H2B	0.9300	C14—H14A	0.9300
C3—C4	1.384 (2)	C15—C16	1.379 (2)
C3—H3A	0.9300	C15—H15A	0.9300
C4—C5	1.393 (2)	C16—C17	1.390 (2)
C4—H4A	0.9300	C16—H16A	0.9300
C5—C6	1.400 (2)	C17—H17A	0.9300
C5—H5A	0.9300		
			
N1—Zn1—N1^i^	104.32 (7)	C7—C8—C9	123.08 (13)
N1—Zn1—S1^i^	121.84 (3)	C7—C8—H8A	118.5
N1^i^—Zn1—S1^i^	86.96 (3)	C9—C8—H8A	118.5
N1—Zn1—S1	86.96 (3)	N1—C9—C8	120.80 (13)
N1^i^—Zn1—S1	121.84 (3)	N1—C9—H9A	119.6
S1^i^—Zn1—S1	134.50 (2)	C8—C9—H9A	119.6
C10—S1—Zn1	92.11 (5)	N2—C10—S2	118.39 (11)
C10—S2—C11	104.17 (7)	N2—C10—S1	130.26 (11)
C9—N1—N2	114.33 (12)	S2—C10—S1	111.35 (8)
C9—N1—Zn1	129.50 (10)	C12—C11—S2	105.99 (10)
N2—N1—Zn1	116.07 (9)	C12—C11—H11A	110.5
C10—N2—N1	113.42 (12)	S2—C11—H11A	110.5
C2—C1—C6	120.33 (14)	C12—C11—H11B	110.5
C2—C1—H1A	119.8	S2—C11—H11B	110.5
C6—C1—H1A	119.8	H11A—C11—H11B	108.7
C1—C2—C3	120.48 (14)	C13—C12—C17	118.62 (14)
C1—C2—H2B	119.8	C13—C12—C11	120.44 (14)
C3—C2—H2B	119.8	C17—C12—C11	120.92 (14)
C4—C3—C2	119.80 (14)	C14—C13—C12	121.01 (15)
C4—C3—H3A	120.1	C14—C13—H13A	119.5
C2—C3—H3A	120.1	C12—C13—H13A	119.5
C3—C4—C5	120.19 (15)	C15—C14—C13	120.01 (16)
C3—C4—H4A	119.9	C15—C14—H14A	120.0
C5—C4—H4A	119.9	C13—C14—H14A	120.0
C4—C5—C6	120.44 (15)	C16—C15—C14	119.63 (15)
C4—C5—H5A	119.8	C16—C15—H15A	120.2
C6—C5—H5A	119.8	C14—C15—H15A	120.2
C5—C6—C1	118.75 (13)	C15—C16—C17	120.35 (15)
C5—C6—C7	119.05 (13)	C15—C16—H16A	119.8
C1—C6—C7	122.18 (13)	C17—C16—H16A	119.8
C8—C7—C6	125.72 (14)	C12—C17—C16	120.37 (15)
C8—C7—H7A	117.1	C12—C17—H17A	119.8
C6—C7—H7A	117.1	C16—C17—H17A	119.8
			
N1—Zn1—S1—C10	−6.84 (6)	C6—C7—C8—C9	−174.96 (14)
N1^i^—Zn1—S1—C10	98.14 (6)	N2—N1—C9—C8	−176.13 (12)
S1^i^—Zn1—S1—C10	−140.35 (5)	Zn1—N1—C9—C8	7.7 (2)
N1^i^—Zn1—N1—C9	64.77 (12)	C7—C8—C9—N1	−178.89 (14)
S1^i^—Zn1—N1—C9	−30.62 (14)	N1—N2—C10—S2	−176.19 (9)
S1—Zn1—N1—C9	−173.11 (13)	N1—N2—C10—S1	3.1 (2)
N1^i^—Zn1—N1—N2	−111.33 (10)	C11—S2—C10—N2	11.23 (14)
S1^i^—Zn1—N1—N2	153.28 (8)	C11—S2—C10—S1	−168.22 (8)
S1—Zn1—N1—N2	10.79 (9)	Zn1—S1—C10—N2	4.50 (14)
C9—N1—N2—C10	172.96 (13)	Zn1—S1—C10—S2	−176.13 (7)
Zn1—N1—N2—C10	−10.34 (15)	C10—S2—C11—C12	171.23 (11)
C6—C1—C2—C3	−0.6 (2)	S2—C11—C12—C13	−84.45 (16)
C1—C2—C3—C4	−0.6 (2)	S2—C11—C12—C17	94.11 (16)
C2—C3—C4—C5	0.8 (2)	C17—C12—C13—C14	1.2 (3)
C3—C4—C5—C6	0.1 (2)	C11—C12—C13—C14	179.80 (16)
C4—C5—C6—C1	−1.2 (2)	C12—C13—C14—C15	−0.8 (3)
C4—C5—C6—C7	−179.72 (14)	C13—C14—C15—C16	−0.1 (3)
C2—C1—C6—C5	1.4 (2)	C14—C15—C16—C17	0.6 (3)
C2—C1—C6—C7	179.91 (14)	C13—C12—C17—C16	−0.7 (3)
C5—C6—C7—C8	−175.41 (15)	C11—C12—C17—C16	−179.28 (16)
C1—C6—C7—C8	6.1 (2)	C15—C16—C17—C12	−0.2 (3)

Symmetry codes: (i) −*x*, *y*, −*z*+1/2.

### Hydrogen-bond geometry (Å, °)

**Table:**

*D*—H···*A*	*D*—H	H···*A*	*D*···*A*	*D*—H···*A*
C13—H13A···S2^ii^	0.93	2.76	3.6697 (17)	167
C11—H11B···Cg1^iii^	0.97	2.98	3.5785 (17)	121

Symmetry codes: (ii) *x*, −*y*+1, *z*+1/2; (iii) *x*+1/2, −*y*+1/2, −*z*+1.
